# Supplementary material for: Transcriptome Analysis Reveals Vimentin-Induced Disruption of Cell–Cell Associations Augments Breast Cancer Cell Migration
Source: Cells. 2022 Dec 13;11(24):4035. doi: 10.3390/cells11244035 (PMC9776984; doi:10.3390/cells11244035)
Supplement: Supplementary file 1 [file cells-11-04035-s001.zip › DEGs and enrichment analysis.zip!/DEGs and enrichment analysis/WT_VIMvsVector KEGG/ALL/WT_VIMvsVector_ALL.pathway_map.zip!/WT_VIMvsVector_ALL.pathway_map/src/hsa04926.html]

hsa04926


- hsa:51764

- Up regulated genes

ENSG00000127588(3.22298284123795)

- hsa:1388

- Up regulated genes

novel.600(2.78845209317737)
- hsa:90993

- Down regulated genes

ENSG00000157613(-3.21711806186639)

- hsa:1906

- Down regulated genes

ENSG00000078401(-3.03994258238563)

- hsa:51764

- Up regulated genes

ENSG00000127588(3.22298284123795)

- hsa:1287

- Down regulated genes

ENSG00000188153(-2.16664393151527)
- hsa:1285

- Down regulated genes

ENSG00000169031(-2.54975234049394)
- hsa:1281

- Up regulated genes

ENSG00000168542(1.50071136748401)

- hsa:4322

- Down regulated genes

ENSG00000137745(-2.44541898505839)

- hsa:51764

- Up regulated genes

ENSG00000127588(3.22298284123795)

- hsa:107

- Up regulated genes

ENSG00000164742(1.87205666251591)

- hsa:51764

- Up regulated genes

ENSG00000127588(3.22298284123795)

- hsa:107

- Up regulated genes

ENSG00000164742(1.87205666251591)

- hsa:107

- Up regulated genes

ENSG00000164742(1.87205666251591)

Close
